# Supplementary material for: Genomic Signatures of SARS-CoV-2 Associated with Patient Mortality
Source: Viruses. 2021 Feb 2;13(2):227. doi: 10.3390/v13020227 (PMC7912856; doi:10.3390/v13020227)
Supplement: Supplementary file 1 [file viruses-13-00227-s001.zip › Supplementary Table 3.pdf]

Supplementary Table 3. Mortality rates among demographic and geographic parameters

		Mortality rate (% [95%CI])	n/N
**Global**		5.74 [4.89-6.72]	144/2508
**Sex***	Male	6.85 [5.61-8.34]	90/1314
	Female	5.41 [4.17-6.99]	54/999
**Geography***	Africa	0.93 [0.22-5.10]	1/107
	Asia	4.51 [3.50-5.80]	57/1264
	Europe	3.84 [2.65-5.52]	27/704
	North America	9.70 [7.28-15.61]	23/214
	South America	19.89 [14.73-26.31]	36/181
**Time***	Dec 2019	0 [0.32-36.94]	0/7
	Jan 2020	0 [0.02-2.86]	0/126
	Feb 2020	0.43 [0.11-2.39]	1/230
	Mar 2020	4.36 [3.22-5.89]	40/917
	Apr 2020	11.44 [9.20-14.15]	73/638
	May 2020	4.88 [2.69-8.75]	10/205
	Jun 2020	10.87 [6.72-17.17]	15/138

*Statistically significant differences in mortality rate according to geography (X^2=61.26, d.f.=4, P<0.0001) and time of year (X^2=74.35, d.f.=6, P<0.0001), but not sex (X^2=2.37, d.f.=1, P=0.12). n: number of deceased, N: total number of patients.
