# Supplementary material for: Genomic Signatures of SARS-CoV-2 Associated with Patient Mortality
Source: Viruses. 2021 Feb 2;13(2):227. doi: 10.3390/v13020227 (PMC7912856; doi:10.3390/v13020227)
Supplement: Supplementary file 1 [file viruses-13-00227-s001.zip › Supplementary Table 4.pdf]

Supplementary Table 4. Parameter estimates of Generalized Linear Model (GLM) for patient mortality

Term	Estimate	Std Error	X^2	P value	Lower CL	Upper CL
Intercept	-6.106	0.521	279.32	<.0001*	-7.243	-5.156
Africa	-1.134	0.676	6.02	0.014*	-2.898	-0.040
Asia	0.121	0.229	3.34	0.067	-0.303	0.639
Europe	-0.544	0.261	5.33	0.021*	-1.046	0.020
North America	0.246	0.290	3.25	0.072	-0.315	0.853
Jan	-1.776	1.260	2.08	0.149	-5.948	0.048
Feb	-1.473	0.775	2.08	0.149	-3.407	-0.103
Mar	-0.133	0.383	1.80	0.179	-0.795	0.783
Apr	0.818	0.376	9.07	0.003*	0.175	1.725
May	0.352	0.447	2.36	0.124	-0.484	1.345
Jun	1.098	0.438	9.24	0.002*	0.300	2.083
Female Sex	-0.190	0.098	8.40	0.004*	-0.388	0.003
Age	0.053	0.006	115.46	<.0001*	0.042	0.065
Clade 2	-0.009	0.176	3.48	0.062	-0.365	0.335
Clade 3	0.571	0.169	14.33	0.0002*	0.236	0.902
Clade 4	-0.253	0.171	5.78	0.016*	-0.597	0.081

*Statistically significant P values. Std Error: standard error. CL: 95% confidence interval of estimates. The overall model had a X^2=180.49, P<0.0001, AICc=809.1, with significant effect tests for geographic region (X^2=48.82, d.f=4, P<0.0001), time of year (X^2=42.39, d.f.=6, P<0.0001, sex (X^2=9.40, d.f.=1, P=0.004), age (X^2=115.46, d.f.=1, P<0.0001) and viral Clades (X^2=22.77, d.f.=3, P<0001).
