# Supplementary material for: Genomic Signatures of SARS-CoV-2 Associated with Patient Mortality
Source: Viruses. 2021 Feb 2;13(2):227. doi: 10.3390/v13020227 (PMC7912856; doi:10.3390/v13020227)
Supplement: Supplementary file 1 [file viruses-13-00227-s001.zip › Supplementary Table 5.pdf]

Supplementary Table 5. Parameter estimates of the Generalized Linear Model (GLM) for patient mortality in the validation cohort

Term	Estimate	Std Error	X^2	P value	Lower CL	Upper CL
Intercept	-7.202	0.998	104.49	<.0001*	-9.5893	-5.353
Africa	-2.850	1.069	16.42	<.0001*	-6.735	-1.210
Asia	0.467	0.430	3.08	0.0792	-0.354	1.596
Europe	0.333	0.434	2.38	0.1231	-0.496	1.452
North America	1.668	0.576	10.03	0.0015*	0.561	2.977
Jan	2.541	1.937	0	1.0000	-2.047	7.138
Feb	0.098	1.517	0	1.0000	-4.174	2.327
Mar	-1.609	0.689	1.96	0.1611	-3.026	-0.248
Apr	0.478	0.564	1.84	0.1746	-0.627	1.662
May	-0.868	0.602	1.95	0.1625	-2.059	0.361
Jun	-0.594	0.568	1.81	0.1784	-1.698	0.599
Age	0.054	0.011	36.10	<.0001*	0.032	0.0776
A23349	-1.359	0.782	4.77	0.0289*	-3.857	0.002
C14353	-0.088	0.357	2.21	0.1368	-0.927	0.541
CA28827-8	-0.637	0.263	8.60	0.0034*	-1.211	-0.112

*Statistically significant P values. Std Error: standard error. CL: 95% confidence interval of estimates. The overall model had a X^2=55.51, P<0.0001. AICc=247.06, with effect tests for geographic region (X^2=29.78, d.f.=4, P<0.0001), time of year (X^2=14.45, d.f.=6, P=0.025, age (X^2=36.10, d.f.=1, P<0.0001), A/G23349 (X^2=4.77, d.f.=2, P=0.0289), C/T14353 (X^2=2.21, d.f.=1, P<0.136, and CA/GG28827-8 (X^2=8.59, d.f.=1, P=0.0034).
